# MicroRNA-383: A tumor suppressor miRNA in human cancer

**DOI:** 10.3389/fcell.2022.955486

**Published:** 2022-10-13

**Authors:** Abdollah Jafarzadeh, Majid Noori, Shaghayegh Sarrafzadeh, Seyed Saeed Tamehri Zadeh, Maryam Nemati, Nazanin Chatrabnous, Sara Jafarzadeh, Michael R Hamblin, Mohammad Hassan Jafari Najaf Abadi, Hamed Mirzaei

**Affiliations:** ^1^ Department of Immunology, School of Medicine, Kerman University of Medical Sciences, Kerman, Iran; ^2^ Immunology of Infectious Diseases Research Center, Research Institute of Basic Medical Sciences, Rafsanjan University of Medical Sciences, Rafsanjan, Iran; ^3^ Golestan Hospital Research Center, AJA University of Medical Sciences, Tehran, Iran; ^4^ Department of Medical Genetics, School of Medicine, Shahid Beheshti University of Medical Sciences, Tehran, Iran; ^5^ School of Medicine, Tehran University of Medical Sciences, Tehran, Iran; ^6^ Department of Immunology, School of Medicine, Rafsanjan University of Medical Sciences, Rafsanjan, Iran; ^7^ Department of Hematology and Laboratory Sciences, School of Para-Medicine, Kerman University of Medical Sciences, Kerman, Iran; ^8^ Endocrinology and Metabolism Research Center, Shiraz University of Medical Sciences, Shiraz, Iran; ^9^ Student Research Committee, School of Medicine, Kerman University of Medical Sciences, Kerman, Iran; ^10^ Laser Research Centre, Faculty of Health Science, University of Johannesburg, Johannesburg, South Africa; ^11^ Department of Medical Biotechnology, School of Medicine, Mashhad University of Medical Sciences, Mashhad, Iran; ^12^ Research Center for Biochemistry and Nutrition in Metabolic Diseases, Institute for Basic Sciences, Kashan University of Medical Sciences, Kashan, Iran; ^13^ Student Research Committee, Kashan University of Medical Sciences, Kashan, Iran

**Keywords:** cancer, miR-383, miR-383-3p, mir-383-5p, target genes

## Abstract

Downregulated expression of anti-tumor miR-383 has been found in many kinds of cancer. MiR-383 family members can directly target the 3′-untranslated region (3′-UTR) of the mRNA of some pro-tumor genes to attenuate several cancer-related processes, including cell proliferation, invasion, migration, angiogenesis, immunosuppression, epithelial-mesenchymal transition, glycolysis, chemoresistance, and the development of cancer stem cells, whilst promoting apoptosis. Functionally, miR-383 operates as a tumor inhibitor miRNA in many types of cancer, including breast cancer, hepatocellular carcinoma, gastric cancer, pancreatic cancer, colorectal cancer, esophageal cancer, lung cancer, head and neck cancer, glioma, medulloblastoma, melanoma, prostate cancer, cervical cancer, oral squamous cell carcinoma, thyroid cancer, and B-cell lymphoma. Both pro-tumor and anti-tumor effects have been attributed to miR-383 in ovarian cancer. However, only the pro-tumor effects of miR-383 were reported in cholangiocarcinoma. The restoration of miR-383 expression could be considered a possible treatment for cancer. This review discusses the anti-tumor effects of miR-383 in human cancers, emphasizing their downstream target genes and potential treatment approaches.

## 1 Background

Noncoding RNAs are divided into several subsets, including circular RNAs (circRNAs), long noncoding RNAs (lncRNAs), and microRNAs (miRNAs) (Dashti et al., 2021; Shafabakhsh et al., 2021; Ahmadpour et al., 2022; Mousavi et al., 2022). MiRNAs are single-stranded non-coding RNAs with 18–25 nucleotides in length, which bind to complementary sequences in the 3′-untranslated region (3′-UTR) of their target mRNAs (Mirzaei and Hamblin, 2020; Razavi et al., 2021). When a miRNA binds to its target mRNA, it can cause mRNA degradation or repress protein translation. MiRNAs can also promote the expression of target proteins by extending the process of mRNA translation (Raue et al., 2021). An individual miRNA may affect the expression of several mRNAs, while several miRNAs could control each mRNA (Chen et al., 2021a; Raue et al., 2021). MiRNAs perform a vital role in controlling cell proliferation, apoptosis, migration, and differentiation by modulating the expression of their target genes (Tamtaji et al., 2021). Dysregulated miRNA expression is linked with tumor growth, progression, and chemoresistance in many cancers (Tu et al., 2019).

LncRNAs are RNA transcripts greater than 200 nucleotides in length that control gene expression by three mechanisms: epigenetically (by triggering histone modification, chromatin remodeling, or DNA methylation), transcriptionally (by binding to transcription factors, gene promoters, or enhancers), or post-transcriptionally (by interacting with miRNAs or by regulating mRNA transport, splicing, and translation) (Alkan and Akgül, 2022). LncRNAs may operate as competitive endogenous RNAs (ceRNAs) to bind to specific miRNAs, and thereby, reduce their activity (Alkan and Akgül, 2022). LncRNAs can play a fundamental role in many biological processes, such as cancer development.

MicroRNA-383 (miR-383) is divided into two subtypes, miR-383-3p and miR-383-5p. The gene of miR-383 is mapped to the chromosome 8p22 region inside intron 3 of the sarcoglycan zeta (SGCZ) gene (Bucay et al., 2017). MiR-383, with the sequence CAC​GAA​AGA​TCA​GAA​GGT​GAT​TG, serves as a tumor inhibitor miRNA in many types of cancer, including breast cancer (BC) (Zhang et al., 2020; Azarbarzin et al., 2021), hepatocellular carcinoma (HCC) (Chen et al., 2016; Fang et al., 2017; Cheng et al., 2020), gastric cancer (GC) (Zhu et al., 2019; Tao et al., 2021), colorectal cancer (CC) (Yan et al., 2018; Sun et al., 2020), pancreatic cancer (PC) (Han et al., 2015; Su et al., 2019), lung cancer (Ma et al., 2016a; Shang et al., 2016), esophageal cancer (Wang et al., 2015; Gao et al., 2020), head and neck cancer (Liu et al., 2019a; Wang et al., 2021), glioma (He et al., 2013; Xu et al., 2014; Xu et al., 2015), medulloblastoma (MB) (Wang et al., 2012; Yuan et al., 2013), melanoma (Pinto et al., 2015; Zhang et al., 2019), prostate cancer (PCa) (Bucay et al., 2017; Huang et al., 2021), cervical cancer (CC) (Hu et al., 2019), oral squamous cell carcinoma (OSCC) (Shao et al., 2020), and B-cell lymphoma (Chen et al., 2021b). Both pro-tumor (Liu et al., 2016) and mainly anti-tumor effects (Vilming Elgaaen et al., 2014; Zhang et al., 2017; Jiang et al., 2019) have been attributed to miR-383 in ovarian cancer. However, only pro-tumor effects of miR-383 were reported in cholangiocarcinoma (Wan et al., 2018a; Wan et al., 2018b). This review discusses the importance of miR-383-3p and miR-383-5p in the development of malignancy while highlighting their downstream target genes and potential therapeutic approaches.

### 1.1 Anti-tumor effects of miR-383 in digestive system cancers

#### 1.1.1 Gastric cancer

In patients with gastric cancer (GC), miR-383 expression was found to be reduced in tumor tissue, which was correlated to a more advanced tumor stage and an unfavorable prognosis (Zhu et al., 2019; Tao et al., 2021). MiR-383 downregulation might be utilized as a diagnostic marker for GC (Azarbarzin et al., 2017). MiR-383-5p expression was shown to be low in GC tissues and cells and it was linked to tumor size and differentiation scores. The survival time was shorter in GC patients with lower expression of miR-383-5p than those with higher levels of miRNA expression (Wei and Gao, 2019). In GC cells, an increase in the expression of miR-383 and miR-383-5p inhibited proliferation and migration, whilst enhancing apoptosis (Wei and Gao, 2019; Xu et al., 2019; Zhu et al., 2019). Using bioinformatics analyses, 49 target genes were identified for miR-383-5p, which contributed to PI3K, TGF-β receptor, mTOR, VEGF/VEGFR, c-Myc, and E-cadherin-related signaling pathways (Wei and Gao, 2019).

Aberrant cellular metabolism has been widely identified in malignant cells, and some drugs have been designed to target cellular metabolism. Lactate dehydrogenase A (LDHA), which is involved in glycolysis, is overexpressed in some tumors, where it is associated with an unfavorable prognosis. There is emerging evidence that suppression of LDHA may exert novel anti-oncogenic effects both by reducing the cellular metabolism and by functioning synergistically with other anti-cancer treatments, such as radiotherapy, cytotoxic chemotherapy, and cancer-targeted drugs (Yang et al., 2021a). LDH is a major enzyme in the aerobic glycolytic pathway, which catalyzes the interconversion of pyruvate and lactate, a reaction involved with NAD+ and NADH interconversion. This tetrameric enzyme has two different subunits: the heart type encoded by LDHB and the muscle type encoded by LDHA (Zhang et al., 2022). In the miR-383-5p/LDHA pathway, LDHA is a target of miR-383-5p. Accordingly, miR-383-5p downregulation results in LDHA overexpression, promoting GC development (Wei and Gao, 2019). In GC tissues, a negative association between miR-383-5p and LDHA expression has been found (Wei and Gao, 2019). Thus, miR-383-5p overexpression can prevent GC development through LDHA repression (Wei and Gao, 2019).

Cyclin E2 has been identified as a major regulator of cell cycle progression through the G1 phase. Previous studies on mouse models have elucidated that cyclin E exerts its effects on mammalian cells through various biologic processes involved in cell division. Healthy cells strictly control the function of cyclin E, which is dysregulated in tumor cells. Furthermore, the abnormal function of cyclin E has been shown to be involved in tumorigenesis (Hwang and Clurman, 2005). In the miR-383/cyclin E2 pathway, cyclin E2 is a downstream target of miR-383. In GC cells, decreased expression of miR-383 resulted in cyclin E2 overexpression. Furthermore, it has been shown that in GC tissues, the expression of miR-383 and cyclin E2 is inversely correlated (Zhu et al., 2019). Cyclin E2 is an important player in the cell cycle in some solid tumors (Milioli et al., 2020). Cyclin E2 activates cyclin-dependent kinase 2 (CDK2) and induces the G1 to the S phase of cell cycle progression (Zhu et al., 2019). Therefore, miR383 upregulation can suppress GC development *via* inhibition of cyclin E2 expression.

Histone deacetylases (HDACs) are a group of histone-modifying enzymes that remove the acetyl group from lysine residues in the amino-terminal tail of histone proteins, and thus, regulate gene transcription. HDACs have a critical role in the regulation of the cell cycle, growth, proliferation, differentiation, and other cellular processes. HDAC expression or function is dysregulated in numerous tumors (Xiong et al., 2019). In the miR-383/HDAC9 pathway, the attenuated expression of miR-383 led to the upregulation of histone deacetylase 9 (HDAC9) in GC tissues and cells, which was related to metastasis, shorter survival times, and unfavorable prognosis in GC patients (Xu et al., 2019). The exogenous expression of miR-383-5p inhibited HDAC9 expression in tumor tissues from GC-bearing nude mice (Xu et al., 2019). HDAC9 interacts with histone and non-histone molecules to regulate transcription in a tissue-specific manner. HDAC9 is overexpressed in cancer cells and affects the carcinogenesis process (Yang et al., 2021b). It can exert pro-tumor or anti-tumor effects depending on the tumor type. The pro-tumor effects of HDAC9 have been demonstrated in breast cancer, oral squamous cell carcinoma, and retinoblastoma (Xu et al., 2019). MiR-383-5p overexpression can suppress GC progression *via* HDAC9 repression (Xu et al., 2019).

Mitochondrial apoptosis is regulated by the counteracting interactions between anti-apoptotic and pro-apoptotic members of BCL-2 family proteins. The balanced relationship between pro-apoptotic and anti-apoptotic BCL-2 proteins regulates programmed cell death and maintains organism health. The dysregulated activity of the BCL-2 family may hinder cellular apoptosis and promote tumor development and resistance to anti-cancer drugs (Campbell and Tait, 2018). In the miR-383/Bcl-2 pathway, decreased miR-383 expression together with elevated Bcl-2 expression was found in GC tissues compared to normal control specimens, which was related to advanced tumor stages and higher metastasis (Tao et al., 2021). In GC specimens, an inverse correlation was found between the expression levels of miR-383 and Bcl-2 (Tao et al., 2021). MiR-383 directly interacted with the 3′-UTR of Bcl-2 to reduce its protein expression, which reduced GC cell proliferation and viability (Tao et al., 2021).

Cancer inhibitor of protein phosphatase 2A (CIP2A) is a well-recognized oncoprotein, which promotes the proliferation of cancer cells, resistance to programmed cell death, and anchorage-independent cell growth. CIP2A inactivates protein phosphatase 2A (PP2A), which reduces the phosphorylation of Akt (protein kinase B) and stabilizes the c-Myc proto-oncogene in tumor cells (Soofiyani et al., 2017a). In the miR-383-5p/CIP2A pathway, decreased miR-383-5p expression together with higher CIP2A expression was found in GC cells compared with normal gastric epithelial cells (Li et al., 2020a). CIP2A promotes cancer cell proliferation and prevents apoptosis (Soofiyani et al., 2017b). MiR-383-5p overexpression restrained GC proliferation, whilst enhancing apoptosis by targeting CIP2A (Li et al., 2020a).

The epidermal growth factor receptor family comprises four receptor types, epidermal growth factor receptor-1 (ErbB1), ErbB2, ErbB3, and ErbB4, all of which are involved in tumorigenesis. ErbB4 is unique among the EGFRs, as it is the only receptor with concurrent growth suppressive effects. ErbB4 has well-recognized roles in the development of normal organ systems, particularly the nervous system, heart, and mammary glands. Recent studies have found that ErbB4 functions as a tumor suppressor in some cancers (Campbell and Tait, 2018). In the miR-383-5p/ERBB4 pathway, attenuated expression of miR-383-5p was accompanied by an increased expression of ERBB4 to promote GC development (Lv et al., 2020). ERBB4 can promote tumor development in some cancers, such as GC (Xu et al., 2018; Segers et al., 2020). In GC cells, allicin (a biologically active compound present in garlic) inhibited cell viability and invasion, whilst promoting apoptosis, which was attributed to miR-383-5p overexpression and ERBB4 downregulation (Lv et al., 2020). Downregulation of ERBB4 led to inhibition of the PI3K/Akt-mediated signaling pathway (Lv et al., 2020). MiR-383-5p can suppress GC development through the repression of ERBB4 expression.

Zinc finger E-box binding homeobox 2 (ZEB2), a DNA-binding transcription factor, is critically involved in the epithelial-to-mesenchymal transition (EMT). EMT is a highly-regulated process through which epithelial cells lose their adhesion and polarity, and then obtain the invasion and migration properties typical of mesenchymal stem cells. Accumulating evidence has shown that ZEB2 has a major role in the EMT process, including growth, differentiation, resistance to therapy and apoptosis, metastatic potential, and tumor recurrence (Fardi et al., 2019). In the miR-383/ZEB2 pathway, low expression of miR-383 caused the upregulation of ZEB2 in gastric MALT lymphoma tissue and cells (Song et al., 2018). In gastric MALT lymphoma tissues, a negative association was also found between the expression levels of miR-383 and ZEB2 (Song et al., 2018). ZEB1 and ZEB2 are transcription factors for marginal zone B cells, which trigger the EMT and increase metastasis in some malignancies (Fardi et al., 2019). In gastric MALT lymphoma cells, miR-383 inhibited cell division by inhibiting ZEB2 expression and acted as a tumor suppressor (Song et al., 2018).

TIPRL is an evolutionarily conserved protein and is a homolog of TIP41 in yeast. In contrast to yeast TIP41, human TIPRL was shown to directly affect protein phosphatase 2A (PP2A), PP6, and PP4, which are all members of the PP2A family of phosphatase enzymes. TIPRL has a major role in the ATM/ATR signal transduction system, which regulates the response to DNA damage, and TOR (target of rapamycin) signaling by controlling PP2A. Previous studies have shown that TIPRL is highly expressed in hepatocellular carcinoma and that the suppression of TIPRL by small interfering RNA induced continuous activity of JNK (c-Jun N-terminal kinase) and MKK7 (mitogen-activated protein kinase 7) by phosphorylation of MKK7. As a result, TIPRL can protect tumor cells from TRAIL (tumor necrosis factor-related apoptosis-inducing ligand)-mediated apoptosis. In the miR-383-5p/TIPRL pathway, the expression of TIPRL in GC tissues was related to the advanced tumor stage, more metastasis, and shorter survival times. MiR-383-5p directly targeted TIPRL mRNA and attenuated its expression (Luan et al., 2020). In GC cells, overexpression of TIPRL reduced cell migration and invasion. TIPRL-induced phosphorylation/activation of AMPK diminished the phosphorylation of 4E-BP1 and p70S6K, inactivated the mTOR-mediated signaling pathway, and subsequently suppressed cell invasion and migration. Therefore, miR-383-5p suppression could upregulate TIPRL, thus preventing GC development (Luan et al., 2020).

#### 1.1.2 Colorectal cancer

MiR-383 expression was shown to be low in CRC tissues, and it was linked to tumor size, metastasis, and tumor stage in CRC patients (Yan et al., 2018). Lower values of miR-383-5p were detected in the serum of CRC patients, which were linked to differentiation, tumor stage, metastasis, and unfavorable prognosis (Sun et al., 2020). In patients with colon cancer, lower values of miR-383 were found in cancer tissue relative to non-malignant tissue, which has an inverse relationship with tumor stage and survival time (Cui et al., 2018). In colon cancer cells, miR-383 overexpression repressed cell division and invasion (Cui et al., 2018). Overexpression of miR-383-5p could sensitize CRC cells to a neo-adjuvant chemotherapeutic drug (Sun et al., 2020).

CREPT (cell cycle-related and expression-elevated protein in tumor) is a regulator of numerous cell cycle proteins and has been shown to affect many biological processes, such as peripheral T-cell activation, differentiation, and metastasis of keratinocytes by controlling genes related to the cell cycle. CREPT may also serve as a tumor-oncogene and may predict the outcome in some tumors. In this regard, CREPT was correlated with an unfavorable prognosis in CRC patients and retroperitoneal leiomyosarcoma (Liang et al., 2017). In the miR-383/CREPT pathway, miR-383 directly interacted with the 3′-UTR of CREPT mRNA, and therefore, reduced CREPT expression. The downregulation of miR-383 was accompanied by the upregulation of CREPT in colon cancer tissues and cells (Li et al., 2018). CREPT potentiated Wnt-mediated signaling by interacting with TCF4 and β-catenin, which led to higher expression of cyclin D1 to support cancer cell proliferation (Li et al., 2018). In colon cancer cells, the forced expression of miR-383 prevented cell proliferation and colony formation, accompanied by the repression of CREPT and related downstream genes (Li et al., 2018). In colon cancer, miR-383 operates as a cancer inhibitor by suppressing CREPT expression (Li et al., 2018).

PAX6 (paired box 6) is an evolutionarily conserved transcription factor and a member of the paired box family. PAX6 is mainly involved in the development of the eyes, nose, central nervous system, and endocrine pancreas. In 2003, the expression of PAX6 mRNA was first shown in various tumor cell lines. The mechanism of action of PAX6 as a tumor suppressor has been shown in retinoblastoma, prostate cancer, and glioblastoma cell lines. On the other hand, some oncogenic properties of PAX6 were found in colorectal adenocarcinoma, retinoblastoma, and breast tumor cell lines (Kiselev et al., 2018). In the miR-383/PAX6 pathway, downregulation of miR-383 together with the upregulation of PAX6 was found in CRC tissues and cells. PAX6 was identified as a direct downstream target for miR-383; therefore, PAX6 showed an inverse relationship with miR-383 expression levels in CRC tissues and cells (Yan et al., 2018). In CRC cells, PAX6 upregulation enhanced cell proliferation, colony formation, and invasion, whilst miR-383 exerted opposite effects by the repression of PAX6 expression (Li et al., 2014; Yan et al., 2018). In CRC, miR-383 can operate as a tumor suppressor by attenuating PAX6 expression.

**FIGURE 1:**
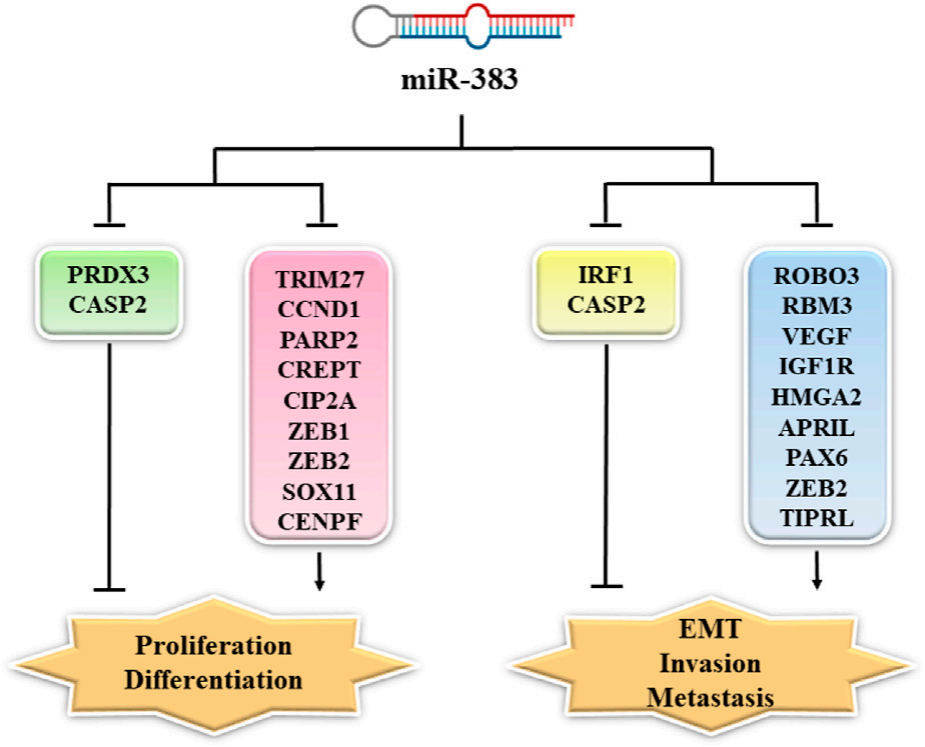
MiR-383 targets and their function in cancer cell proliferation, EMT, invasion, and metastasis. MiR-383 overexpression suppressed cell proliferation *via* inhibition of the expression of CCND1, CREPT, ZEB-1, and others, while it increased proliferation by inhibiting the expression of PRDX3. Moreover, ROBO3, RBM3, VEGF, and other genes are regulated by miR-383 resulting in the suppression of cancer cell invasion and metastasis. MiR-383 also regulates the expression of Caspase-2 and promotes cancer cell invasion and metastasis. PRDX3: peroxiredoxin 3; CASP2: Caspase 2; TRIM27: tripartite motif-containing protein 27; CCND1: cyclin D1; PARP2: poly [ADP-ribose] polymerase 2; CREPT: cell cycle-related and expression-elevated protein in tumor; CIP2A: cancerous inhibitor of protein phosphatase 2A; ZEB1: zinc finger E-box binding homeobox 1; SOX11: Sry-related high-mobility group box 11; ZEB2: zinc-finger E-box binding homeobox 2; CENPF: centromere protein F; IRF1: interferon (IFN) regulatory factor 1; ROBO3: Roundabout; RBM3: RNA-binding motif protein 3; VEGF: vascular endothelial growth factor; IGF1R: insulin-like growth factor 1; HMGA2: high-mobility-group protein AT-hook 2; APRIL: *A* proliferation-inducing ligand; PAX6: paired box 6; TIPRL: target of rapamycin signaling pathway regulator-like; EMT: epithelial–mesenchymal transition.

Members of the TNF (tumor necrosis factor) family are involved in tumorigenesis. Unlike members of the TNF superfamily, APRIL (a proliferation-inducing ligand, TALL-2, TNFSF13) facilitates tumor cell growth. APRIL stimulates the growth, differentiation, maturation, and survival of B cells in normal physiologic conditions. In addition, it is involved in the pathogenesis of various autoimmune diseases, including rheumatoid arthritis and systemic lupus erythematosus. APRIL upregulates the production of IgA by stimulating the TACI receptor (Nowacka and Jabłońska, 2021). In the miR-383/APRIL pathway, miR-383 directly targeted APRIL. In colon cancer cells, the lower expression of miR-383 led to overexpression of APRIL (Cui et al., 2018). In CRC cells, overexpression of APRIL increased proliferation and migration whilst reducing apoptosis (Wang et al., 2013; Cui et al., 2018). In CRC cells, the enforced expression of miR-383 inhibited cell migration and proliferation by attenuating APRIL expression (Cui et al., 2018). Figure 1 shows some gene targets of miR-383 and their function in cancer cell proliferation, EMT, invasion, and metastasis (Yi et al., 2022).

CREB1 (cAMP responsive element binding protein 1) is a member of the bZIP (basic leucine zipper) family and is a transcription factor that modulates the transcription of both upstream and downstream genes. CREB1 binds to the CRE (cyclic-AMP response element) on the promoter of target genes and stimulates corresponding gene expression. Dysregulation of CREB1 was found in numerous tumors, such as breast cancer, acute myeloid leukemia, renal cancer, and non-small cell lung cancer. As a proto-oncogene, CREB1 plays a major role in various tumor processes, including proliferation, metastasis, and invasion. Previous studies have shown that CREB1 regulates the expression of some tumor-related genes, such as tumor necrosis factor-α (TNF-α), c-fos, and Bcl- (Fang et al., 2016). In the circ_0136666/miR-383/CREB1 axis, circ_0136666 serves as an upstream sponge of miR-383 and CREB acts as the downstream miR-383 target. In the CRC cells, circ_0136666-induced miR-383 downregulation caused CREB1 overexpression (Li et al., 2020b). In CRC patients, abnormal overexpression of circ_0136666 and CREB1 was found in CRC tissues and cells, which was associated with tumor size, tumor stage, and metastasis (Li et al., 2020b). CREB1 functions as an oncogene in some cancers, such as CRC. In CRC cells, CREB1 upregulation increased the expression of glycolysis-related proteins, such as lactate dehydrogenase A (LDHA) and hexokinase 2 (HK2), promoting proliferation. In CRC, miR-383 can operate as a cancer suppressor by modulating CREB1 expression (Li et al., 2020b).

#### 1.1.3 Hepatocellular carcinoma

MiR-383 expression was lower in HCC cells and tissues and showed an inverse relationship with tumor size and metastasis (Chen et al., 2016; Fang et al., 2017; Cheng et al., 2020). In patients with HCC, the expression of miR-383 could be considered an independent prognostic marker, and reduced miR-383 expression was linked with shorter survival time (Chen et al., 2016). Furthermore, miR-383 overexpression in HCC cells halted proliferation, while promoting apoptosis (Chen et al., 2016; Cheng et al., 2020).

PHF8 (plant homeodomain finger protein 8) is a recognized proto-oncogene, involved in the formation and progression of tumors (Lv et al., 2017). PHF8 belongs to the family of histone demethylase enzymes and also serves as a transcriptional co-activator. PHF8 binds to the promoter region of about one-third of the human genome and induces transcription by reducing histone markers, such as H4K20me1, H3K27me2, and H3K9me1/2. Accordingly, aberrant expression of PHF8 is associated with the development of various human tumors. For instance, overexpression of PHF8 plays a major role in the development and metastasis of breast cancer, prostate cancer, esophageal squamous cell carcinoma, leukemia, lung cancer, and gastric adenocarcinoma (Zhou et al., 2018). In the miR-383/PHF8 pathway, the miR-383 expression was reduced, whilst the expression of PHF8 was increased in HCC cells and tissues compared with normal samples (Cheng et al., 2020). PHF8 is a histone demethylase enzyme, which is overexpressed in many malignant tumors, such as colorectal, lung, and gastric cancers (Lv et al., 2017; El-Aarag et al., 2017; Li et al., 2017). In HCC cells, miR-383 overexpression or PHF8 silencing inhibited proliferation and invasion. PHF8 is a downstream target of miR‐383; thus, miR-383 is able to suppress the invasion and proliferation of HCC cells by repressing PHF8 expression (Cheng et al., 2020).

Accumulating evidence suggests a pivotal role of the IL-17 family (among other inflammatory cytokines) in tumorigenesis. In addition to IL-17A, numerous clinical trials have implicated the function of the IL-17B/IL-17 receptor B (IL-17RB) pathway in tumor formation and chemoresistance. IL-17B activates 17RB, which results in enhanced tumor cell survival, migration, proliferation, and chemoresistance in mouse models. Moreover, IL-17B signaling can significantly modify the tumor microenvironment by increasing cytokine and chemokine secretion, leading to enhanced tumor progression (Bastid et al., 2020). In the miR-383/IL-17 pathway, miR-383 was downregulated, whilst IL-17 was overexpressed in HCC tissues relative to adjacent control samples (Wang et al., 2019a). In HCC cells, apoptosis was promoted by miR-383, whereas it was suppressed by IL-17 (Wang et al., 2019a). IL-17 exerts pro-tumor effects in HCC, and IL-17 upregulation has been associated with unfavorable prognosis in HCC patients (Liao et al., 2013). It has been demonstrated that IL-17 acts as a downstream target of miR-383; thus, miR-383 operates as an HCC inhibitor and apoptosis enhancer by targeting IL-17 (Wang et al., 2019a).

In the miR-383/LDHA pathway, LDHA was shown to be a target of miR-383, and an inverse relationship was found between the expression of miR-383 and LDHA in HCC tissues (Fang et al., 2017). In HCC cells, LDHA increased glycolysis, proliferation, and invasion, which were all suppressed by miR-383 overexpression (Fang et al., 2017). LDHA performs a vital role in the glycolysis process in cancer, promotes cell proliferation, viability, invasion, angiogenesis, and metastasis, and allows cancer cells to evade the immune response (Feng et al., 2018). In HCC, miR-383 functions as a tumor suppressor by controlling LDHA expression (Fang et al., 2017).

In the miR-383/APRIL pathway, the TNF family member APRIL is a downstream target gene of miR-383 (Chen et al., 2016). In HCC tissues, an inverse association was found between miR-383 and APRIL expression; therefore, miR-383 can suppress HCC, partly by repressing APRIL expression (Chen et al., 2016).

EIF5A2 (eukaryotic translation initiation factor 5A1) and its associated homolog eIF5A2 are the only human proteins that undergo specific hypusination, a post-translational modification that is necessary for their function. Hypusine is a rare amino acid synthesized by two enzymes, deoxyhypusine synthase and deoxyhypusine hydroxylase. EIF5A2 is a translation initiation factor involved in the formation of the first peptide bond during protein translation and can also act as an elongation factor during translation. Recent studies have shown that EIF5A2 is involved in some human diseases, such as diabetes and numerous cancers as a tumor-promoting factor (Wu et al., 2020). In the miR-383/EIF5A2 pathway, miR-383 overexpression and EIF5A2 silencing sensitized HCC cells to doxorubicin-induced cytotoxicity and apoptosis, whilst miR-383 inhibition exerted opposite effects. In HCC cells, EIF5A2 overexpression promoted EMT, proliferation, and invasion (Ning et al., 2020). In HCC patients, a reverse correlation was found between the expression of EIF5A2 and survival time (Ning et al., 2020). EIF5A2 is a miR-383 target, and miR-383 overexpression reduced doxorubicin resistance in HCC cells by suppressing EIF5A2 expression (Tu et al., 2019).

Aldo-keto reductase family 1 member B10 (AKR1B10) also operates as an oncogene in HCC; so, in HCC cells, AKR1B10 silencing inhibited cell proliferation (Wang et al., 2018a). AKR1B10 metabolizes xenobiotics and removes some endogenous carbonyl compounds, thereby promoting chemoresistance in cancer cells (Endo et al., 2021). AKR1B10 is overexpressed in several types of cancer, such as HCC (Endo et al., 2021). MiR-383-5p is an upstream regulator targeting AKR1B10 and represses its expression (Wang et al., 2018a).

In the PTTG3P/miR-383/CCND1-PARP2 axis, the lncRNA pituitary tumor-transforming 3 (PTTG3P) sponges miR-383, thereby attenuating the inhibitory effects of this miRNA on cyclin D1 (CCND1) and poly ADP-ribose polymerase 2 (PARP2) expression (Zhou et al., 2019). In HCC tissues, PTTG3P was upregulated and its expression levels were positively correlated with tumor stage, size, and metastasis (Zhou et al., 2019). In HCC cells, silencing of PTTG3P inhibited invasion and proliferation, whilst increasing apoptosis. In HCC cells, PTTG3P overexpression increased PARP2 and CCND1 expression, whereas miR-383 overexpression exerted opposite effects. Both PARP2 and CCND1 act as oncogenes, which support HCC growth by activating the PI3K and Akt-related signaling pathways (Zhou et al., 2019). In the PTTG3P/miR-383/CCND1-PARP2 axis, PTTG3P acts as a sponge of miR-383 to enhance the expression of PARP2 and CCND1, along with the PI3K/Akt pathway, which potentiates HCC development. PARP2 and CCND1 are direct downstream targets of miR-383; thus, miR-383 overexpression exerts inhibitory effects on HCC development *via* CCND1 and PARP2 repression (Zhou et al., 2019).

In the HULC/miR-383-5p/VAMP2 axis, the expression of lncRNA was highly upregulated in liver cancer (HULC), and the expression of vesicle-associated membrane protein-2 (VAMP2) was enhanced, whereas miR-383-5p was downregulated in HCC tissues (Li et al., 2021). In HCC cells, the upregulation of HULC promoted cell expansion, inhibited apoptosis, and enhanced chemoresistance to oxaliplatin. However, the elevated expression of miR-383-5p (a direct target of HULC) eliminated the HULC effects on HCC proliferation and chemo-resistance to oxaliplatin (Azarbarzin et al., 2021). The expression of VAMP2 showed a reverse relationship with the expression of miR‐383‐5p in HCC tissues. HULC can act as a sponge of miR-383-5p to promote the expression of VAMP2. HULC enhanced HCC progression and chemoresistance to oxaliplatin by modulating the miR-383-5p/VAMP2 pathway (Azarbarzin et al., 2021).

#### 1.1.4 Pancreatic cancer

Remarkable miR-383 downregulation was found in PC tissues and cells relative to control samples (Han et al., 2015). In PC patients, intratumoral downregulation of miR-383 was associated with tumor progression and poor prognosis (Su et al., 2019). In human PC cells, increased expression of miR-383 prevented cell division while increasing apoptosis (Su et al., 2019).

Gab (Grb2-associated binding proteins) such as Gab1, Gab2, and Gab3 in mammalian cells act as scaffold proteins in protein–protein interactions and also act as amplifiers of signal transduction in several pathways involved in tissue maturation. Recent studies have clarified their role in the formation and progression of some cancers. For example, Gab2 upregulation is closely correlated with progression and metastasis in malignant melanoma, breast cancer, and ovarian tumors. In addition to Gab2, Gab1 was significantly associated with progression, proliferation, and metastasis in colorectal cancer and squamous cell carcinoma of the head and neck (Wang et al., 2019b). In the miR-383/GAB1 pathway, low expression of miR-383 results in the upregulation of GAB1, thereby promoting PC development (Su et al., 2019). The pro-tumorigenesis effects of GAB1 were also found in several cancers, such as melanoma and CRC. In PC cells, downregulation of GAB1 attenuated cell migration and proliferation, while inducing apoptosis. MiR-383 can directly interact with GAB1 to inhibit its expression. MiR-383 suppressed PC development and progression by downregulating GAB1 expression (Su et al., 2019).

Previous studies have shown that the Slit glycoprotein and Roundabout (Robo) signaling pathway may be involved in the progression of numerous cancers. Therefore, the Slit/Robo signaling pathway has emerged as a possible target in cancer research. Approximately 5–15% of patients with pancreatic cancer have been found to have mutated Slit and/or Robo genes, and up to 48% of cases show abnormal methylation of the Slit or Robo gene, suggesting that abnormal signaling in this pathway may contribute to the pathogenesis of PC. Increased activity of the WNT/*β*-catenin signaling pathway, which is a conserved pathway involved in determining cell fate, was also found in PC, and this pathway could be regulated by the Slit/Robo pathway (Ding et al., 2020). In the miR-383/ROBO3 pathway, miR-383 directly interacts with ROBO3; thus, miR-383 operates as a repressor of ROBO3 expression. In PC tissues, downregulation of miR-383 caused ROBO3 overexpression; thus, miR-383 and ROBO3 showed an inverse relationship (Han et al., 2015). Overexpression of ROBO3 potentiated tumorigenesis through the promotion of EMT and the induction of Wnt, β-catenin, and GSK-3-related pathways. In PC, overexpression of miR-383 could suppress tumor progression by the suppression of ROBO3 expression (Han et al., 2015).

The SRY (sex-determining region Y)-box11 (SOX11) gene has a crucial role in embryonic neurogenesis, cell differentiation, and tissue remodeling (Sock et al., 2004). SOX11 may play distinct roles in various types of cancer, so it shows an increase in some cancers and a decrease in others (Liu et al., 2019b). SOX11 prevents the proliferation and invasion of cancer cells (Chen et al., 2010). It promotes apoptosis and G2/M-phase cell cycle arrest in human hepatoma HuH-7 cells (Liu et al., 2019b). Studies have confirmed that SOX11 is a direct target of miR-383. Likewise, Wnt is the target gene of miR-383. It was found that miR-383 inhibits the progression of pancreatic carcinoma by targeting SOX11. Moreover, overexpression of miR-383 or inhibition of Wnt expression suppressed cell viability and induced apoptosis in cancer cells. It seems that the mir-383/SOX11 axis affects the Wnt/β-catenin signaling pathway, thereby reducing its activation and further inducing apoptosis, inhibiting the viability of cancer cells and reducing tumorigenic capacity (Wang et al., 2018b; Liu et al., 2019b). Xue et al. also recently demonstrated the role of lncRNA TMPO-AS1 (lncRNA TMPO antisense RNA 1) as a tumor promoter by regulation of the miR-383/SOX11 axis. They indicated that lncRNA TMPO-AS1 promotes cell proliferation, migration, and invasion of pancreatic carcinoma by sponging miR-383-5p and upregulating SOX11.

#### 1.1.5 Esophageal cancer

In esophageal squamous cell carcinoma (ESCC) cells, miR-383 up-regulation prevented proliferation, while promoting apoptosis (Wang et al., 2015). In the miR-383/5S rRNA pathway, 5S rRNA functions as a direct target of miR-383. In ESCC cells, the decreased expression of miR-383 resulted in 5S rRNA upregulation. The 5S rRNA was overexpressed in the ESCC tissues and a reverse correlation was found between the miR-383 and 5S rRNA expression levels (Wang et al., 2015). 5S rRNA enhances tumor development *via* several mechanisms, such as repression of P53 expression and prevention of MDMX degradation. 5S rRNA silencing also strengthens the interaction of rpL11 with c-Myc, which attenuated the oncogenic activity of c-Myc (Wang et al., 2015). Moreover, miR-383 downregulates CCND1 expression by affecting the 5S rRNA-rpL11-c-Myc pathway and by direct binding to CCND1 mRNA (Wang et al., 2015).

In the FGD5-AS1/miR-383/SP1 axis, miR-383 functions as the downstream target of a lncRNA called FGD5 antisense RNA 1 (FGD5-AS1); thus, miR-383 can be sponged by FGD5-AS1 (Gao et al., 2020). Moreover, miR-383 directly targets specificity protein 1 (SP1); thus, its expression is attenuated by miR-383. In patients with ESCC, FGD5-AS1 overexpression displayed a strong relationship with tumor size, stage, and metastasis. Shorter overall survival was found in ESCC patients who had upregulation of FGD5-AS1 relative to those with low levels of FGD5-AS1 (Gao et al., 2020). In ESCC tissues and cells, upregulation of FGD5-AS1 led to SP1 overexpression by repression of miR-383. SP1 supports tumor progression by enhancing angiogenesis, proliferation, invasion, and reducing apoptosis (Vellingiri et al., 2020). Therefore, miR-383 overexpression suppressed ESCC progression *via* SP1 downregulation (Gao et al., 2020).

### 1.2 Anti-tumor effects of miR-383 in urogenital cancers

#### 1.2.1 Prostate cancer

It has been shown that PCa tissues express lower levels of miR-383-5p than normal prostate tissues (Bucay et al., 2017; Huang et al., 2021). In PCa patients, low miR-383 expression in cancer tissues is related to an unfavorable prognosis and higher serum levels of PSA (Bucay et al., 2017). Anti-metastatic effects of miR-383 were also found in an experimental metastatic PCa model (Bucay et al., 2017).

CD44 is a non-kinase glycoprotein located in the transmembrane space and is highly expressed in tumor stem cells. CD44 has additional splice variants that are significantly involved in tumor formation and progression. Hyaluronan is a major CD44 ligand, which binds to and stimulates CD44, leading to the induction of intracellular signaling pathways that promote cell proliferation, motility, survival, and intracellular cytoskeleton modifications (Chen et al., 2018). In the miR-383/CD44 pathway, miR-383 directly regulates CD44 expression, a common marker of PCa stem cells (Bucay et al., 2017). Low miR-383 expression levels in CD44^+^ PCa cells suggested that miR-383 could regulate cancer stem cell development. In CD44^+^ PCa stem cells, miR-383 overexpression led to CD44 downregulation; thus, miR-383 has the potential to regulate CD44 expression (Bucay et al., 2017). MiR-383 operates as a suppressor of PCa stem cell development by directly suppressing CD44 expression (Bucay et al., 2017).

In the SNHG1/miR-383-5p pathway, miR-383-5p was found to be a downstream target of the lncRNA small nucleolar RNA host gene 1 (SNHG1). In PCa cells, SNHG1 sponges miR-383-5p to increase cell migration and proliferation, while reducing apoptosis. Accordingly, SNHG1 silencing or miR-383-5p overexpression suppressed PCa development (Huang et al., 2021).

#### 1.2.2 Cervical cancer

In the LINC01128/miR-383-5p/SFN axis, the upregulation of LINC01128 and Stratifin (SFN) was accompanied by miR-383-5p downregulation in CC tissues and cells and was related to tumor development and poor prognosis. MiR-383-5p is a downstream target of LINC01128 and can be sponged by this lncRNA, while SFN was found to be a downstream miR-383-5p target (Hu et al., 2019). LINC01128 promotes SFN activity by sponging miR-383-5p, which leads to CC development. SFN exerts pro-carcinogenesis effects through mechanisms such as promoting cell proliferation, cancer stem cell development, EMT, and inhibition of apoptosis (Hu et al., 2019). In CC cells, overexpression of miR-383-5p showed anti-tumor effects by inhibiting SFN expression (Hu et al., 2019).

#### 1.2.3 Ovarian cancer

Experimental studies have shown that miR-383 expression was lower in OC tissues and cells (Zhang et al., 2017). The attenuated expression of miR-383 was found in malignant tissues of patients suffering from high-grade and clear cell OC (Vilming Elgaaen et al., 2014). In the OC cells, increased miR-383 expression blocked OC cell proliferation and invasion, whilst miR-383 downregulation showed opposite effects (Zhang et al., 2017; Jiang et al., 2019). In OC cells, miR-383-5p overexpression also enhanced chemosensitivity (Jiang et al., 2019).

TRIM27 (tripartite motif-containing 27) is a member of the zinc finger superfamily and comprises a tripartite motif containing a coiled-coil domain, B-box zinc finger, and RING finger. It was initially called the RET (REarranged during transfection) proto-oncogene, encoding a receptor with tyrosine kinase activity, but it is also referred to as the RET finger protein (RFP). TRIM27 showed a tumor-promoting effect in NIH3T3 mouse embryonic cell lines when the tripartite domain and the tyrosine kinase portion of the RET proto-oncogene become combined through the rearrangement of DNA. It was shown that TRIM27 can be detected in central and peripheral neurons, male germ cells, liver hepatocytes, and chromaffin cells of the adrenal gland. However, it is also upregulated in several cancer cells, including breast cancer, endometrial cancer, and lung cancer. Moreover, abundant TRIM27 expression has been associated with migration, proliferation, and sensitivity to chemotherapy in some tumors and may, therefore, act as a prognostic marker in some tumors (Ma et al., 2016b). In the miR-383-5p/TRIM27 pathway, TRIM27 functions as a target of miR-383-5p, and the expression of TRIM27 was inversely modulated by miR-383-5p (Jiang et al., 2019). In OC tissues, downregulation of miR-383-5p was detected, along with the upregulation of TRIM27. Overexpression of TRIM27 in tumor tissues promotes tumor progression (Zoumpoulidou et al., 2012; Ma et al., 2016a). In OC cells, miR-383-5p upregulation suppressed cell proliferation and increased sensitivity to chemotherapy by inhibiting TRIM27 expression (Jiang et al., 2019).

In the miR-383/LDHA pathway, LDHA serves as a direct miR-383 target; thus, lower miR-383 expression causes LDHA upregulation. In OC tissues, an inverse relationship was found between the expression levels of LDHA and miR-383 (Zhang et al., 2017). In cellular metabolism, the formation of lactate from pyruvate is catalyzed by LDHA (Zhang et al., 2017), which is characteristic of cancer glycolysis. Therefore, miR-383-5p overexpression can prevent OC development by LDHA suppression.

The oncogenic activity of miR-383 was also reported in OC. In the miR-383/CASP2 pathway, the caspase-2 (CASP2) gene has been identified as a miR-383 target in epithelial ovarian cancer (EOC) (Liu et al., 2016). In EOC cells, the increased expression of miR-383 downregulates CASP2 expression (Liu et al., 2016). In human EOC, the repression of miR-383 could exert tumor-suppressor effects by upregulating CASP2 expression.

### 1.3 Anti-tumor effects of miR-383 on head and neck neoplasms

#### 1.3.1 Head and neck squamous cell carcinoma

In patients with head and neck squamous cell carcinoma (HNSCC), the miR-383 expression level was lower and was related to an unfavorable prognosis (Liu et al., 2019a). In the MIR4435-2HG/miR-383-5p/RBM3 axis, upregulation of MIR4435-2HG and RNA-binding motif protein 3 (RBM3) together with the downregulation of miR-383-5p all contribute to HNSCC development. In patients with HNSCC, upregulation of MIR4435-2HG was associated with an unfavorable prognosis and tumor metastasis (Wang et al., 2021). In HNSCC cells, MIR4435-2HG silencing blocked cell proliferation, invasion, and EMT. In HNSCC cells, MIR4435-2HG serves as an upstream sponge of miR-383-5p, and this miRNA directly targets the 3′-UTR of RBM3 mRNA to repress its expression. Thus, MIR4435-2HG sponges miR-383-5p to cause RBM3 overexpression and promote tumor progression. Moreover, an *in vitro* study showed that miR-383-5p overexpression abrogated the tumor-promoting effects of MIR4435-2HG by repressing RBM3 expression (Wang et al., 2021).

In the HOXC13-AS/miR-383-3p/HMGA2 axis, the overexpression of HOXC13 antisense RNA (HOXC13-AS) and high mobility group A2 (HMGA2) and the downregulation of miR-383-3p were found in nasopharyngeal carcinoma (NPC) tissues and cells (Gao et al., 2019). Here, miR-383-3p is the downstream target of HOXC13-AS and can be sponged by this lncRNA (Gao et al., 2019). However, HMGA2 serves as a downstream target of miR-383-3p; thus, the expression of HMGA2 is directly downregulated by miR-383-3p (Gao et al., 2019). HOXC13-AS indirectly promotes HMGA2 activity by sponging miR-383-3p, which promotes the development of NPC. HMGA2 promotes carcinogenesis through mechanisms, such as inducing cancer cell expansion, cancer stem cell development, and apoptosis inhibition (Mansoori et al., 2021). In NPC cells, both HOXC13-AS inhibition and miR‐383‐3p up-regulation blocked cell proliferation and invasion, whilst increasing apoptosis. In NPC cells, overexpression of miR-383-3p can exert anti-tumor effects by suppressing HMGA2 expression (Gao et al., 2019).

#### 1.3.2 Oral squamous cell carcinoma

In the RP11-284F21.9/miR-383-5p/MAL2 axis, the upregulation of lncRNA RP11-284F21.9, and MAL2, accompanied by the downregulation of miR-383-5p, was found in OSCC tissues and cells (Shao et al., 2020). MAL2 is a direct miR-383-5p target. RP11‐284F21.9 directly sponges miR‐383‐5p, leading to the upregulation of MAL2, which supports OSCC progression (Shao et al., 2020). In OSCC cells, RP11-284F21.9 silencing decreased MAL2 expression, proliferation, and migration (Shao et al., 2020). MAL2 is a transmembrane protein, which is overexpressed in some cancers, such as ovarian, colorectal, and breast cancers. Thus, miR-383-5p overexpression could prevent OSCC progression by blocking MAL2 expression (Shao et al., 2020).

### 1.4 Anti-tumor effects of miR-383 in breast cancer

Breast cancer (BC) is the most frequent cancer among women worldwide (Jafari et al., 2021). The expression of miR-383-5p was decreased in BC tissues and cells compared to control tissues and normal epithelial cells (Zhang et al., 2020). Markers of BC progression, including lymph node metastasis and tumor stage, were inversely linked with miR-383-5p expression levels (Zhang et al., 2020; Azarbarzin et al., 2021). Shorter survival times were observed in BC patients who expressed lower miR-383-5p levels than those expressing greater amounts of miR-383-5p (Zhang et al., 2020). The enforced expression of miR-383-5p inhibited BC cell proliferation, colony formation, viability, and invasion, while miR-383-5p downregulation exerted opposite effects (Zhang et al., 2020; Azarbarzin et al., 2021). Moreover, miR-383-5p overexpression increased apoptosis accompanied by the upregulation of pro-apoptosis factors, such as caspase-3, caspase-9, and BAX, and the downregulation of the anti-apoptosis agent Bcl-2. It has been demonstrated that in BC cells, miR-383-5p overexpression was associated with lower values of metastasis-related gene expression, including vimentin, MMP-2, MMP-3, and MMP-9 (Azarbarzin et al., 2021). Moreover, miR-383 overexpression in BC cells increased apoptosis in response to UV or cisplatin by targeting DNA-damage-inducible 45 gamma (GADD45g) (Zhao et al., 2014).

The expression of PD-L1 (programmed cell death ligand 1) was increased and miR-383-5p was decreased in BC tissues compared to normal tissues. PD-L1, a co-inhibitory factor of the immune response, engages with programmed cell death-1 (PD-1) in immune cells to reduce their proliferation and induce apoptosis. The PD-1/PD-L1 axis plays a vital role in cancer immune escape and has a great impact on cancer therapy (Han et al., 2020; Liu et al., 2022). The elevated expression of PD-L1 was related to an unfavorable prognosis in BC patients (Zhang et al., 2021). In BC, miR-383-5p directly targets PD-L1 and prevents cancer progression (Dong et al., 2018). The miR-383-5p overexpression decreased PD-L1 expression. Co-culture of T cells with BC cells revealed that miR-383-5p promoted the expression of INF-γ, IL-2, and TNF-α, while reducing the expression of IL-10 and TGF-β, indicating the ability of miR-383-5p to block tumor-induced immunosuppression.

The expression of PI3K, AKT, and mTOR was reduced in miR-383-5p-transfected BC cells, indicating that miR-383-5p acts as a tumor inhibitor by suppressing the PI3K/AKT/mTOR axis (Azarbarzin et al., 2021). The suppressive effects of miR-383-5p on the PI3K/AKT/mTOR axis can reduce the intratumoral expression of PD-L1 (Azarbarzin et al., 2021). The PI3K/AKT/mTOR-related signaling pathway and PD-L1 expression are powerfully suppressed by miR-383-5p, which inhibits BC development and tumor-induced immunosuppression (Azarbarzin et al., 2021).

In the miR-383-5p/LDHA pathway, lactate dehydrogenase A (LDHA) has been identified as a direct downstream miR-383-5p target (Zhang et al., 2020). LDHA is a glycolytic enzyme catalyzing lactate formation from pyruvate. In addition to normal cell metabolism, LDHA acts as a promotor in cancer development (Yetkin-Arik et al., 2019). An increase in the expression of miR-383-5p inhibited BC cell proliferation and invasion by targeting LDHA (Zhang et al., 2020).

In the circ_0001791/mir-383-5p pathway, circ_0001791 can target mir-383-5p to enhance BC cell invasion and proliferation *via* induction of the PI3K/AKT-dependent signaling pathway (Ameli-Mojarad et al., 2021). Elevated expression of circ_0001791 was found in BC tissues compared with paired adjacent control tissues (Ameli-Mojarad et al., 2021).

In the LINC00096/miR-383-5p/RBM3 axis, lncRNA LINC00096 was upregulated in the triple-negative BC (TNBC) tissues, which was correlated with tumor stage, invasion, and the unfavorable prognosis of patients (Tian et al., 2019). In TNBC cells, LINC00096 inhibition reduced proliferation and invasion (Tian et al., 2019). LINC00096 directly sponged miR-383-5p to upregulate the expression of RNA-binding motif protein 3 (RBM3). RBM3 can act as a proto-oncogene by promoting cell proliferation and preventing apoptosis (Zhou et al., 2017). RBM3 overexpression promoted EMT, whilst miR-383-5p mimics inhibited the EMT process. The overexpression of miR-383-5p can lead to the inhibition of BC *via* suppression of RBM3 expression (Tian et al., 2019).

### 1.5 Anti-tumor effects of miR-383 in lung cancer

It has been shown that mir-383 exerts inhibitory effects on lung cancer, and reduced expression of mir-383 was observed in lung cancer tissues and cells (Ma et al., 2016a; Shang et al., 2016). Decreased expression of miR-383 and miR-383-5p was associated with more advanced stages of lung cancer than early-stage malignancy (Shang et al., 2016; Zhao et al., 2017a). In patients who were diagnosed with small cell lung cancer, low miR-383 expression in tumor tissues was related to advanced TNM stages, more metastasis, and an unfavorable prognosis (Shang et al., 2016). In small cell lung cancer cells, miR-383 overexpression blocked cell division and migration (Shang et al., 2016). Increased expression of miR‐383 in lung squamous cell carcinoma patients (LUSC) was linked with higher survival rates (Neizer-Ashun and Bhattacharya, 2021). In a lung cancer xenograft model, miR-383 overexpression inhibited tumorigenesis and cancer cell invasion (Ma et al., 2016a). An *in vitro* study showed that miR-383-5p overexpression was able to block proliferation, whilst inducing apoptosis (Zhao et al., 2017a).

CHEK1 (cell cycle checkpoint kinase 1) is a highly-conserved protein kinase commonly found in fusion yeast, serving as a major regulator of cell cycle progression. CHEK1 is encoded by a gene located on the fourth region of the long arm of human chromosome 11 (11q24). This gene is composed of 13 exons, with the cDNA having a length of 1891 bp, and encoding a protein with a molecular weight of 54 kD. Mutated genes (deletions, base modifications, or DNA rearrangement) affect cellular proliferation and protein expression and subsequently cause tumorigenesis. This may arise from defects in DNA repair, regulatory pathways of apoptosis, and cell cycle disruption. CHEK1 was found in previous studies to act as a major regulator of cell cycle progression and its upregulation can lead to the development of several human cancers, including colorectal, lung, stomach, cervical, and gastric (Wu et al., 2019). In the miR‐383/CHEK1 pathway, CHEK1 was overexpressed in LUSC tissues, and miR‐383 was found to regulate CHEK1 expression (Qi et al., 2019). CHEK1 plays an important role in DNA replication and cell cycle progression. In some malignancies, CHEK1 upregulation was related to cancer progression, tumor recurrence, and resistance to treatment (Neizer-Ashun and Bhattacharya, 2021). Upregulation of miR‐383 can prevent lung cancer growth by suppressing CHEK1 expression (Qi et al., 2019).

In the mir-383-5p/TMPO-AS1 pathway, lower expression of miR-383-5p resulted in the upregulation of lncRNA TMPO antisense RNA 1 (TMPO-AS1) in lung adenocarcinoma tissues and cells. In lung cancer patients, TMPO-AS1 overexpression was shown to be associated with an unfavorable prognosis. In lung cancer cells, TMPO-AS1 silencing inhibited proliferation, suppressed invasion, and promoted apoptosis, whilst upregulation of TMPO-AS1 exerted opposite effects. (Mu et al., 2020). TMPO-AS1 is a direct target of mir-383-5p, and a negative association was found between mir-383-5p and TMPO-AS1 levels in LUAD samples (Mu et al., 2020). MiR-383-5p upregulation can suppress lung cancer cell development by targeting TMPO-AS1.

In the miR-383-5p/CIP2A pathway, CIP2A was identified as a downstream target of miR-383-5p. A negative relationship was found between miR-383-5p and CIP2A expression levels in lung cancer cells and tissues (Zhao et al., 2017a). In lung cancer cells, inhibition of CIP2A led to reduced protein levels of c-Myc, enhanced apoptosis, and decreased cell proliferation (Nader et al., 2019). CIP2A exerted oncogenic effects by inducing c-Myc, JNK, and AKT-related signaling pathways in lung cancer (Nader et al., 2019). MiR-383-5p exerts anti-proliferative effects on lung cancer cells by targeting CIP2A (Zhao et al., 2017a).

EPAS1 (endothelial PAS domain-containing protein 1) has a 48% sequence identity compared to HIF-1α and regulates the interactions between EGFR and MET hepatocyte growth factor receptor. EPAS1 was found to be involved in several cancer types. For example, the transcription activity of EPAS1 was controlled by methylated CpG binding protein 3 in breast cancer, which ultimately modulated cellular responses to EPAS1. The knockdown of EPAS1 could reduce tumor burden and the formation of new blood vessels in pancreatic adenocarcinoma. EPAS1 transcription is controlled by DNA methylation, which could be a prognostic indicator. In non-small cell lung cancer, EPAS1 bound directly to EGFR with a TKI-resistant T790M mutation; however, it did not bind to the wild-type EGFR. The interaction between T790M EGFR and EPAS1 can promote MET signaling in the absence of EGF ligand binding. EPAS1 may function as a link between stimulation of MET and T790M EGFR, indicating that this protein may be a potential treatment target, particularly in non-small cell lung cancers resistant to TKIs (Zhen et al., 2021). In the miR‐383/EPAS1 pathway, miR‐383 prevents lung cancer development by targeting the 3′-UTR of EPAS1 mRNA (Ma et al., 2016a). In lung cancer cells, EPAS1 overexpression abrogated the inhibitory effects of miR-383 on cell expansion. In lung cancer patients, EPAS1 overexpression in malignant tissues was related to an unfavorable prognosis (Ma et al., 2016a).

In the circ-CCS/miR-383/E2F7 axis, upregulation of circ-RNA copper chaperone for superoxide dismutase (circ-CCS is also called circ_0092306) and E2F transcription factor 7 (E2F7) along with miR-383 downregulation in lung cancer tissues have all been shown to be related to advanced tumor stage and unfavorable prognosis (Yuan et al., 2021). Circ-CCS functions as an upstream sponge for miR-383, while E2F7 serves as a downstream target of miR-383. In lung cancer cells, upregulation of circ-CCS resulted in the overexpression of E2F7, which promotes cell expansion. Circ-CCS sponges miR-383, thus, removing the inhibitory effects of miR-383 on E2F7 expression, leading to E2F7 overexpression (Yuan et al., 2021). In lung cancer cells, the silencing of circ-CCS led to the upregulation of miR-383, which suppressed cell proliferation, promoted apoptosis, and prevented tumorigenesis (Yuan et al., 2021). In lung cancer, the anti-tumor effects of miR-383 could be partly explained by E2F7 suppression (Yuan et al., 2021).

### 1.6 Anti-tumor effects of miR-383 in brain tumors

Glioblastoma is the most common malignancy among primary brain tumors (Sisakht et al., 2022). Human glioma tissues and cells showed a lower expression of miR-383 relative to normal controls (He et al., 2013; Xu et al., 2014; Xu et al., 2015). In glioma patients, overexpression of miR-383 was associated with lower tumor grades (He et al., 2013; Xu et al., 2014; Xu et al., 2015). In glioma cells, upregulation of miR-383 prevented cell invasion and proliferation, and enhanced apoptosis (He et al., 2013; Xu et al., 2014; Xu et al., 2015).

The family of cyclin D proteins controls the progression of the cell cycle, a process regulated by interaction with cyclin-dependent kinases 2, 6, and 4. Up to now, three different isoforms have been identified, namely, CCND1 (cyclin D1), CCND2 (cyclin D2), and CCND3 (cyclin D3). Upregulation of CCND1 is associated with tissue differentiation, metastasis, and poor survival in cancer patients. Abundant expression of CCND1 was shown to be correlated with non-small cell lung cancer, pancreatic carcinoma, and squamous cell carcinoma of the head and neck. CCND1, CCND3, and CCND2 have different roles in cancer depending on cell and tissue types. Overexpression of CCND2 was detected in testicular and ovarian cancer, and its abundant expression promoted the progression of gastric adenocarcinoma (Shan et al., 2017). The oncogene CCND1 was identified as a direct miR-383 target in the miR-383/CCND1 pathway (Xu et al., 2014). Lower expression of miR-383 resulted in the overexpression of CCND1 in glioma cells, promoting the transition from G0/G1 to the S phase, and therefore, increasing proliferation (Xu et al., 2014).

In the miR-383/CIP2A pathway, miR-383 directly targets CIP2A (Zhang and Wang, 2020). In glioma tissues and cells, miR-383 downregulation causes the upregulation of CIP2A, which was linked with larger tumor size, higher grade, and shorter survival time in patients (Zhang and Wang, 2020). In glioma cells, CIP2A overexpression promoted cell proliferation and invasion. CIP2A binds to protein phosphatase 2A (PP2A) to prevent PP2A-induced dephosphorylation of c-Myc, increasing c-Myc stability and potentiating AKT activity (Elgendy et al., 2019; O'Connor et al., 2018). Overexpression of miR-383 reduced CIP2A expression in glioma cells, thereby suppressing tumor growth (Zhang and Wang, 2020).

IGF1R (insulin-like growth factor 1 receptor) is a complex and highly regulated signaling pathway for the proliferation and survival of cells. The IGF-IGF1R axis is composed of three receptors with intrinsic tyrosine kinase activity: IGF1R, IGF2R (insulin-like growth factor-2 receptor), and INSR (insulin receptor). The ligands that bind to these receptors include insulin, IGF-1 (insulin-like growth factor-1), IGF-2 (insulin-like growth factor-2), and IGFBPs (serum insulin-like growth factor binding proteins). IGF-1 and IGF-2 have endocrine, paracrine, and autocrine functions and trigger the IGF1R signaling pathway. These growth factors and their corresponding receptors are highly expressed in malignant tumors. This overexpression promotes proliferation, metastasis, invasion, and resistance to anti-cancer drugs and inhibits apoptosis (Yuan et al., 2018). In the miR-383/IGF1R pathway, miR-383 directly targets IGF1R (He et al., 2013). In glioma cells, low expression of miR-383 causes IGF1R to be upregulated. Subsequently, in the IGF1R/AKT/MMP2 axis, IGF1R stimulates AKT, which enhances MMP2 production, resulting in increased invasion (He et al., 2013). Moreover, IGF1R performs key roles in several malignant processes, including transformation, proliferation, viability, apoptosis inhibition, angiogenesis, invasion, and metastasis (He et al., 2013; Werner et al., 2021).

In the lnc-NLC1-C/miR-383/PRDX3 axis, the overexpression of lncRNA narcolepsy candidate region 1 gene C [lnc-NLC1-C, also known as LINC00162] and peroxiredoxin 3 (PRDX3) accompanied by miR-383 downregulation promoted cell proliferation and invasion, while inhibiting apoptosis and autophagy in glioma cells (Xu et al., 2021a). PRDX3 was identified as a direct target gene of miR-383 (Yuan et al., 2013; Xu et al., 2021a). Lnc-NLC1-C overexpression upregulated PRDX3 due to miR-383 suppression. The primary function of PRDX3 is to detoxify reactive oxygen species (ROS) (Yuan et al., 2013). To guarantee the elimination of large amounts of harmful ROS generated during rapid proliferation, PRDX3 levels in tumor cells are higher. PRDX3 expression is elevated in cancers of the breast, lung, liver, and prostate (Yuan et al., 2013).

In the TMPO-AS1/miR-383-5p pathway, miR-383-5p functions as a direct target of lncRNA TMPO antisense RNA1 (TMPO-AS1). In glioma cells, overexpression of TMPO-AS1 together with the low expression of miR-383-5p has been linked with proliferation and migration (Liu et al., 2020). Inhibition of TMPO-AS1 and increased expression of miR-383-5p inhibited glioma progression.

Moreover, glioma-exposed endothelial cells (GECs) expressed lower amounts of miR-383 than normal endothelial cells (Zhao et al., 2017b). Vascular endothelial growth factor (VEGF) is the most important pro-angiogenesis agent in glioma and is directly targeted by miR-383 [16, 17]. Increased expression of miR-383 in GECs inhibited migration, proliferation, and angiogenesis by blocking VEGF expression (Zhao et al., 2017b). VEGF/VEGFR2-induced FAK and Src signaling pathways can promote the malignant behavior of GECs. Therefore, miR-383 exerts inhibitory effects on GECs by repressing VEGF/VEGFR2-stimulated FAK and Src pathways (Zhao et al., 2017b).

Remarkable miR-383 downregulation was found in medulloblastoma (the most prevalent brain tumor in children) tissues and cells (Wang et al., 2012; Yuan et al., 2013). It was shown that in MB cells, miR-383 upregulation blocked proliferation and decreased the expression of Bcl‐XL and Bcl‐2 while enhancing apoptosis (Yuan et al., 2013). High PRDX3 expression was found in MB tumors compared with normal tissues (Wang et al., 2012; Yuan et al., 2013). MiR-383 operates as a tumor suppressor in MB, in part by targeting PRDX3 (Yuan et al., 2013). In MB cells, PRDX3 can promote proliferation and viability and inhibit apoptosis (Yuan et al., 2013). In MB cells, the increased expression of miR-383 inhibited proliferation and enhanced apoptosis by repressing PRDX3 expression (Xu et al., 2021a). MiR-383 can also interact with lnc-NLC1-C to control PRDX-3 expression (Xu et al., 2021a).

In the circSKA3/miR-383-5p/FOXM1 axis, circSKA3 [circ_0029696, named circSKA3 for spindle and kinetochore-associated complex subunit 3 gene (SKA3)] and forkhead box M1 (FOXM1) were overexpressed, while miR-383-5p was underexpressed in MB tissues (Wang et al., 2020). CircSKA3 acts as a sponge for miR-383-5p, and FOXM1 is a direct miR-383-5p target. In MB cells, circSKA3 can sponge miR-383-5p to promote proliferation and invasion, while inhibiting apoptosis (Wang et al., 2020). In MB cells, miR-383-5p upregulation suppressed cell invasion and proliferation and promoted apoptosis by repressing FOXM1 expression (Wang et al., 2020). The transcription factor FOXM1 interacts with the promoter region of various target genes to promote the growth of malignancies (Kalathil et al., 2020; Wang et al., 2020).

### 1.7 Anti-tumor effects of miR-383 in melanoma

The centromere protein F (CENPF) is a cell cycle-associated nuclear antigen that is expressed at low levels in G0/G1 cells but accumulates in the nuclear matrix during the S-phase, with maximal expression in G2/M cells. CENPF has been identified as a marker of cell proliferation in several human cancers, including BC, and was overexpressed in HCC and other tumors. Additionally, elevated CENPF expression contributed to unregulated cell proliferation in HCC. It was recently shown that CENPF and FOXM1 are synergistic master regulators of prostate cancer progression and are prognostic indicators for poor survival and metastasis (Sun et al., 2019). Concerning the miR-383-5p/CENPF pathway, low miR-383-5p expression and overexpression of CENPF in melanoma cells led to increased proliferation and migration (Xu et al., 2021b). In melanoma cells, miR-383-5p overexpression suppressed CENPF expression (Xu et al., 2021b). In melanoma tissues, miR-383-5p expression was inversely correlated with ENPF expression (Shahid et al., 2018). CENPF exerts pro-tumor effects in several malignancies. For instance, CENPF and FOXM1 promote invasion, progression, and drug resistance in PCa in a synergistic manner (Aytes et al., 2014; Lin et al., 2016). MiR-383-5p directly targets CENPF mRNA; therefore, miR-383-5p acts as a tumor inhibitor in melanoma (Xu et al., 2021b).

In human epidermoid carcinoma cells, STAT3 overexpression promoted anti-apoptosis gene expressions, such as Mcl-1, Bcl-1, and STAT3 depletion, making cells susceptible to apoptosis (Liao et al., 2015). STAT3 upregulates the expression of the ataxia telangiectasia-mutated and Rad3-related (ATR) in epidermoid carcinoma cells. ATR promotes cell cycle arrest and DNA repair, and inhibitors of ATR could be promising anti-cancer therapeutic agents (Mei et al., 2019). In epidermoid carcinoma cells, miR-383 reduced ATR expression by targeting the 3′-UTR of ATR mRNA (Liao et al., 2015). However, STAT3 reduced miR-383 expression (Liao et al., 2015). The clarification of the miR-383/ATR pathway in cancer requires more research.

### 1.8 Anti-tumor effects of miR-383 in thyroid cancer

The expression of AKT3 mRNA was reduced in thyroid cancer tissues, and an inverse relation was found between the expression of AKT3 and miR-338-3p. The oncogene AKT3 acts as a direct miR-383-3p target, thus increasing miR-383 expression, suppressing AKT3 expression, and blocking AKT3-related downstream pathways in TC cells (Sui et al., 2017).

### 1.9 Anti-tumor effects of miR-383 in B-cell lymphoma

The miR-383-5p expression was lower in diffuse large B-cell lymphoma (DLBCL) tissues and cells than in control samples. In DLBCL cells, miR-383-5p upregulation inhibited cell expansion and invasion (Chen et al., 2021b). The prognostic value of miR-383-5p in patients suffering from DLBCL was established in clinical studies. Increased levels of miR-383-5p expression could predict a good clinical outcome in DLBCL patients (Chen et al., 2021b).

### 1.10 Anti-tumor effects of miR-383 in cholangiocarcinoma

Increased miR-383 expression was reported in cholangiocarcinoma tissues and cells, which was correlated with tumor size, cancer stage, and poor prognosis (Wan et al., 2018a).

IRF1 (interferon regulatory factor 1) belongs to the IRF family and is activated by several cytokines, including TNF-α, IFN-γ, and interleukins 1–6, which play a major role in controlling the expression of other cytokines such as IL-5, IL-4, IL-13, and IL-12 and govern the activity of immune cells, including Th1 and Th9 lymphocytes. In addition, previous studies have found that variations in the responsiveness of IRF1 to IFN-γ may result in different clinical results. IRF1 was shown to play a critical role in the development of several types of cancer, such as cervical, breast, hepatocellular, prostate, colorectal, and pancreatic ductal adenocarcinoma (Xu et al., 2021c). In the miR-383/IRF1 pathway, IRF1 is a functional miR-383 target, so a negative correlation was found between the expression levels of IRF1 and miR-383 in cholangiocarcinoma tissues (Wan et al., 2018a). In cholangiocarcinoma cells, miR-383 overexpression enhanced cell invasion and proliferation by inhibiting IRF1 (Wan et al., 2018a). IRF1 acts as a tumor inhibitor in cancers, including cholangiocarcinoma (Alsamman and El-Masry, 2018). In cholangiocarcinoma patients, lower IRF1 expression in tumor tissues was correlated with tumor progression and poor prognosis. MiR-383 supports cholangiocarcinoma cell expansion and migration by inhibiting IRF1 expression (Wan et al., 2018a; Wan et al., 2018b). Accordingly, the downregulation of miR-383 resulted in IRF1 overexpression, which inhibited cholangiocarcinoma progression.

## 2 Conclusion

MiR-383 functions as a tumor inhibitor miRNA, and lower miR-383 expression is a common characteristic of many types of cancer (Table 1, Table 2). Accordingly, the enforced expression of miR-383 could have therapeutic potential to prevent cancer development by suppressing various tumor-promoter elements (Figure 1). MiR-383 may exert pro-tumor effects in cholangiocarcinoma (Wan et al., 2018a; Wan et al., 2018b). Therefore, modulation of miR-383 expression could inhibit this malignancy. Notably, single-cell transcriptomics allows quantitative measurement of the molecular activity that underlies the phenotypic multiplicity of cells within a tumor and offers tremendous opportunities to better understand cancer pathogenesis, heterogeneity, and microenvironmental interactions to provide a foundation for new therapeutic innovations (Fan et al., 2020). In this context, the upstream epigenetic and transcriptomic elements that control miR-383 expression need to be further clarified in future studies. The clarification of the prognostic and diagnostic applications of miR-383 in malignancies requires more research.

**TABLE 1:** Anti-tumor effects of miR-383 and its tumor promoter target genes in various types of malignancies.

Cancer type	MiR-383 expression	MiR-383 target genes	Reference
Breast cancer	Downregulated	GADD45g	Zhao et al. (2014)
		PD-L1*	Zhang et al. (2021)
		LDHA	Zhang et al. (2020)
		RBM3*	Tian et al. (2019)
Gastric cancer	Downregulated	LDHA*	Wei and Gao, (2019)
		Cyclin E2	Zhu et al. (2019)
		HDAC9	Xu et al. (2019)
		Bcl-2	Tao et al. (2021)
		CIP2A*	Soofiyani et al. (2017b)
		ERBB4*	Lv et al. (2020)
		ZEB2	Song et al. (2018)
		TIPRL*	Luan et al. (2020)
Colorectal cancer	Downregulated	CREPT	Li et al. (2018)
		PAX6	Yan et al. (2018)
		APRIL	Cui et al. (2018)
		CREB1	Mirzaei and Hamblin, (2020)
Hepatocellular carcinoma	Downregulated	PHF8	Cheng et al. (2020)
		IL-17	Qu et al. (1987)
		LDHA	Fang et al. (2017)
		APRIL	Chen et al. (2016)
		EIF5A2	Ning et al. (2020)
		AKR1B10	Wang et al. (2018a)
		CCND1	Zhou et al. (2019)
		PARP2	Zhou et al. (2019)
		VAMP2*	Shafabakhsh et al. (2021)
Pancreatic cancer	Downregulated	GAB1	Su et al. (2019)
		ROBO3	Han et al. (2015)
Lung cancer	Downregulated	CHEK1	Qi et al. (2019)
		TMPO-AS1*	Mu et al. (2020)
		CIP2A*	Zhao et al. (2017a)
		EPAS1	Ma et al. (2016a)
		E2F7	Yuan et al. (2021)
Esophageal cancer	Downregulated	5S rRNA	Wang et al. (2015)
		SP1	Gao et al. (2020)
		CCND1	Gao et al. (2020)
Head and neck cancer	Downregulated	RBM3*	Wang et al. (2021)
		HMGA2**	Gao et al. (2019)
Glioma	Downregulated	CCND1	Xu et al. (2014)
		CIP2A	Zhang and Wang, (2020)
		IGF1R	He et al. (2013)
		PRDX3	Zhao et al. (2013)
Medulloblastoma	Downregulated	PRDX3	Xu et al. (2021a)
		FOXM1*	Wang et al. (2020)
Melanoma	Downregulated	MACC1**	Zhang et al. (2019)
		CENPF*	Xu et al. (2021b)
		ATR	Liao et al. (2015)
Prostate cancer	Downregulated	CD44	Bucay et al. (2017)
Cervical cancer	Downregulated	Stratifin*	Hu et al. (2019)
Oral cancer	Downregulated	MAL2*	Shao et al. (2020)
Thyroid cancer	Downregulated	AKT3**	Sui et al. (2017)
Ovarian cancer	Downregulated	TRIM27*	Jiang et al. (2019)
		LDHA	Han et al. (2017)

The symbols * and ** indicate that the target gene is targeted by miR-383-5p and miR-383-3p, respectively.

**TABLE 2:** Upstream pro-tumor modulators of miR-383 in various cancers.

Cancer type	LncRNA regulator	LncRNA expression	Target	Reference
Breast cancer	LINC00096	Upregulated	miR-383-5p	Tian et al. (2019)
	circ_0001791	Upregulated	miR-383-5p	Ameli-Mojarad et al. (2021)
Colorectal cancer	circ_0136,666	Upregulated	miR-383	Mirzaei and Hamblin, (2020)
Hepatocellular carcinoma	PTTG3P	Upregulated	miR-383	Zhou et al. (2019)
	HULC	Upregulated	miR-383-5p	Shafabakhsh et al. (2021)
Lung cancer	circ_CCS	Upregulated	miR-383	Yuan et al. (2021)
Esophageal cancer	FGD5-AS1	Upregulated	miR-383	Gao et al. (2020)
Head and neck cancer	MIR4435-2HG	Upregulated	miR-383-5p	Wang et al. (2021)
	HOXC13-AS	Upregulated	miR-383-3p	Gao et al. (2019)
Glioma	lnc-NLC1-C	Upregulated	miR-383	Zhao et al. (2013)
	TMPO-AS1	Upregulated	miR-383-5p	Liu et al. (2020)
Medulloblastoma	circSKA3	Upregulated	miR-383-5p	Wang et al. (2020)
Prostate cancer	SNHG1	Upregulated	miR-383-5p	Huang et al. (2021)
Cervical cancer	LINC01128	Upregulated	miR-383-5p	Hu et al. (2019)

## Glossary

APRIL: A proliferating-inducing ligand
BCL-2: B-cell lymphoma-2
CASP2: Caspase 2
CCND1: Cyclin D1
CDK2: Cyclin-dependent kinase 2
CIP2A: Cellular inhibitor of PP2A
c-MYC: MYC proto-oncogene
DIO1: Iodothyronine deiodinase 1
EIF5A2: Eukaryotic translation initiation factor 5A2
EMT: Epithelial-to-mesenchymal transition
EPAS1: Endothelial PAS domain protein 1
ERBB4: Erb-b2 receptor tyrosine kinase 4
FAK: Focal adhesion kinase
FOXM1: Forkhead box M1
GAB1: GRB2-associated binding protein 1
GADD45g: Growth arrest and DNA damage inducible gamma
GDNF: Glial cell-derived neurotrophic factor
H2AX: H2AX variant histone
HDAC9: Histone deacetylase 9
IRF1: Interferon regulatory factor 1
LDHA: Lactate dehydrogenase A
MALAT1: Metastasis-associated lung adenocarcinoma transcript 1
MiRNAs: microRNAs
MSCs: Mesenchymal stem cells
NSCLC: Non-small-cell lung cancer
PARP: Poly (ADP-ribose) polymerase
PARP2: poly (ADP-ribose) polymerase 2
PAX6: Paired box 6
PD-L1: Programmed cell death Ligand 1
PNUTS: Protein phosphatase 1 regulatory subunit 10
PP2A: Protein phosphatase 2 phosphatase activator
PRDX3: Peroxiredoxin 3
ROBO3: Roundabout guidance receptor 3
RPRD1B: Regulation of nuclear pre-mRNA domain containing 1B
SOX11: SRY-box transcription factor 11
SP1: Sp1 transcription factor
TGF-β1: Transforming growth factor β 1
TRIM27: Tripartite motif containing 27
TNF-α: Tumor necrosis factor-α
VEGF: Vascular endothelial growth factor
WNT1: Wnt family member 1
ZEB2: Zinc finger E-box binding homeobox 2
